# Correlates of Cognitive Impairment among Indian Urban Elders

**DOI:** 10.4172/2167-7182.1000489

**Published:** 2018-11-06

**Authors:** Poojitha Reddy Konda, Pawan Kumar Sharma, Atul R Gandhi, Enakshi Ganguly

**Affiliations:** 1 Mediciti Institute of Medical Sciences, Ghanpur, Hyderabad, India; 2 Department of Community Medicine, Mediciti Institute of Medical Sciences, Ghanpur, Hyderabad, India; 3 Department of Epidemiology, University of Pittsburgh, and Share India, Fogarty International NIH, USA; 4 Consultant Statistician & Chief Manager-Monitoring and Evaluation, EdelGive Foundation, Edelweiss House, Mumbai, India

**Keywords:** Cognitive impairment, Dementia, Elderly, Correlates, Prevalence, Urban India

## Abstract

**Background:**

Cognitive impairment among elderly is increasing owing to increases in life expectancy globally. The problem is multifactorial. The objective of the present paper was to study the correlates of cognitive impairment in an urban elderly population in India.

**Methods:**

A cross sectional study was conducted among 100 randomly selected urban elderly population. Data was collected upon household visits using a predesigned pretested questionnaire administered by a trained investigator. Measurements included cognitive function assessment using Mini Mental State Examination, depression assessment using Geriatric Depression Scale, blood pressure measurement and anthropometry. Cognitive impairment was defined at MMSE score <24. Logistic regression was done to identify independently associated factors with cognitive impairment.

**Results:**

Prevalence of cognitive impairment among elderly was 10%. Women had a higher prevalence than men. Higher age, no schooling, living single, lower weight, lower waist and hip ratios, difficulty in activities of daily living, poor self-reported health, bedridden and depression significantly associated with cognitive impairment. The independently associated factors upon logistic regression were increasing age, no schooling and bedridden status for past six months.

**Conclusion:**

Although the current prevalence of cognitive impairment among Indian urban elderly is low, several associated factors exist in this population that may increase the burden in future. Geriatric health policy should address the modifiable risk factors to manage the problem of cognitive impairment and its consequent outcomes.

## Introduction

Global population of elderly is growing at a fast pace. India, the projected second highest contributor to world’s elderly after China, will be home for 323 million elderly population (60 years or older) by the year 2050 compared to current 93 million in the year 2011 [1]. The current demographic transition leading to increases in older population [2], is likely to present challenges such as poor quality of life and increased dependency [3] primarily due to several changes in ageing brain affecting (and including) cognition [4]. Independent studies clearly showed cognitive function decline with increasing age [5,6]. Cognition is crucial to human thinking and experiences, referring to a process of identifying, selecting, interpreting, storing, and using information to make sense of and interact with the physical and social world, to conduct one’s everyday activities, and to plan and enact the course of one’s occupational life. Cognition has been shown as an important factor for independent living and measure of brain health of the population [7]. Researchers from western countries have reported that physical health of the elderly that include mobility disability, falls, fractures, fatigue, frailty, cardiovascular events, obesity, constipation and others, are related to their cognitive state [8–14].

Previous research reported a 15.7% prevalence of cognitive impairment (10.5% mild impairment, 5.2% moderate to severe impairment) among urban population aged 60 year old and above [15]. Older age, race, fewer years of education, likelihood of cardiovascular disease and undernutrition were shown to be positively associated with cognitive impairment. The same study predicted increased hospitalizations, visits to emergency departments and greater mortality amongst those progressing to dementia. Among other risk factors, some have found impairment in global cognition, attention, memory, visuospatial performance, or frontal-executive function among hypertensive elderly [16,17]. Orthostatic hypotension, in particular, has been shown to be associated with decreased cerebral blood flow [18,19], therefore suggesting its role in cognitive impairment. These findings underline the importance of regular physical activity as a potent protective factor for cognitive decline and dementia in elderly persons [20].

We found a paucity of studies describing the prevalence and correlates for cognitive impairment among Indian elderly. The Lucknow Elders Study, amongst the very few from India, reported a prevalence of 7.6% cognitive impairment measured objectively [21]. Elsewhere, prospective studies have shown a high burden, with the prevalence of cognitive impairments doubling every 5 years, affecting 20% of those aged 65 years and 45% of those aged ≥90 years [22]. In this research paper, we attempted to report the functional changes of the brain measured by cognitive function of urban Indian elderly. The objective of this research paper was to determine correlates of cognitive impairment in south Indian urban elders.

## Research Methodology

### Study design and participants

This cross-sectional analysis was done using data from a cross-sectional study conducted in later half of year 2016, with the primary objective to assess brain health of the Urban Elders, aged 60 years and above, in Hyderabad city, a south-central part of India. Data was collected from 100 subjects (52% men and 48% women) out of 124 eligible subjects contacted from 10 townships selected using population proportion to size method (amidst approximately 30 listed townships), adequately representing Hyderabad city. We enlisted the households with age eligible elders and randomized households in each township. All age eligible subjects from the selected households were contacted to participate in the study. The participation rate was 94.33%. The eligibility criteria were: men and women aged 60 years and above, apparently healthy, residing in urban residential townships, possessing ability to understand the investigators’ instructions in English, Hindi or Telugu languages and provided consent to be included in the study. We excluded those who had known neurodegenerative disease, had psychosis or taking psychotropic medication, or living in a nursing home or assisted-care facility. Ethics approval was obtained from the Institutional Ethics Committee of MediCiti Institute of Medical Sciences. All participants provided written consent on a locally translated informed consent form.

The questionnaires and forms were designed using questionnaires adapted from WHO Study of Global AGEing and Adult Health (SAGE) [23], Health Aging and Body Composition study (Health ABC) [24] and Mobility and Independent Living in Elders Study (MILES) [25] and it was pretested upon 20 peri-urban elderly individuals before administrating. Construct validity of the tool was ensured by blind back translation into English from local language.

Data was collected on age and sex of the participants; socio-demographic characteristics including years of schooling, occupation, marital status; self-reported general health, addictions, medical history, comorbidities, functional disabilities, depression, sleep, cognitive function, blood pressure, anthropometry and medication.

Each participant completed an interview and clinical examination in approximately 40 ± 23 minutes. The responses were recorded by a trained investigator who administered the questionnaire to the participant, maintaining privacy. Clear instructions were given to the participants for each step as per the standard protocol. If participant failed to understand instructions, the investigator demonstrated the procedure.

### Definitions and measurements

#### Cognitive function:

Persons with cognitive impairment had trouble with verbal recall, basic arithmetic, and concentration. We measured orientation, registration, attention and calculation, recall and language and praxis using Mini Mental State Examination (MMSE) scale [26].

We classified cognitive impairment and dementia based on Mini Mental State Examination (MMSE): as a score<24 was defined as cognitive function impairment; scores<21 was defined as dementia while scores between 24 – 30 implied no cognitive impairment, 18 – 23 as mild cognitive impairment and 0 – 17 as severe cognitive impairment [27,28]

#### Depression:

Depression was measured using Geriatric Depression Scale (GDS) (15point scale) [29]. Subjects scoring > 5 were defined as having depressive symptoms.

#### Quality of sleep:

Quality of sleep was measured by Pittsburgh Sleep Quality Index (PSQI). subjects having scores ≥ 5 were categorised having poor sleep quality [30].

#### Activities of daily living (ADL):

Activities of daily living (ADLs) included difficulty in walking across a small room, bathing, eating, dressing, moving in and out of bed and using toilet. If the participant had difficulty in performing one or more out of these six activities without assistance was considered to have difficulty in ADL [31].

#### Poor hearing and vision:

Hearing was defined as poor in a person who self-reported poor hearing or complete deafness. Vision was described as self-reported, poor or very poor vision.

#### Anthropometry:

Height was measured in centimetres using Seca 214 stadiometer (Seca, Hanover, MD). Weight was measured in kilograms and grams using a Seca digital platform scale (Seca 813 Digital scale) with very light clothing. Waist circumference and hip circumference was measured for each participant using a non-flexible fibreglass tape to nearest 1 mm. A single thin layer of clothing was allowed during hip circumference measurements in view of local dressing norms in order not to violate the dignity of the participants. Waist hip ratio and BMI (kg/m^2^) was calculated.

#### Blood pressure:

Resting blood pressure and heart rate measured seated participants after an initial resting period of 5 minutes. Systolic and diastolic blood pressure was recorded in the left arm in sitting position, using digital monitor, Omron Hem-705 (Omron Healthcare, Inc., Lake Forest, IL). An average of two readings was taken as the final systolic or diastolic blood pressure of the individual.

### Statistical methods

We analysed data using SPSS 21.0 (SPSS Inc., Chicago, IL, USA). We reported characteristics and correlates of cognitive impairment and no cognitive impairment among subjects as mean (± standard deviation, SD) and proportions. Categorical variables were compared using chi-square test and continuous variables by t-test. The variables which were statistically significant (p<0.05) on univariate analysis were considered for multivariate model and adjusted to age and sex. Specifically, we entered sex, age (per 1 SD increase), weight (per 1 SD increase), waist circumference (per 1 SD increase), hip circumference (per 1 SD increase), marital status, education, self-reported health status, depression, activities of daily living (ADL) and bed ridden in the multivariate model. For continuous variables we expressed OR with 95% confidence limits (95% CI) into per 1 SD increase. We excluded difficulty in moving around from the model, as it was highly collinear with ADL. Backward logistic regression was performed (employing deletion of each variable using a chosen model comparison criterion, deleting the variable with poor fit for model improvement and repeating this process until no further improvement was possible) for multivariate analysis to get final set of the independent risk factor variables. The variables having p<0.05 (considered significant) were reported as independent risk factors of cognitive impairment. The results of logistic regression were reported as odds ratios (OR) with 95% confidence interval (95% CI).

## Results

### Background characteristics of population

52% of the respondents were men. There was no difference in ages of women and men. (Table 1) Women were significantly more non-educated and living single than men. Their mean height weight, waist circumference and BMI were significantly different than men (p<0.05).

### Prevalence of cognitive impairment

Total prevalence of cognitive impairment was 10%. Cognitive impairment was greater among women (6%) than men (4%) (p=0.06). The mean MMSE score was greater for men compared with women although the difference was not significant. Prevalence of dementia (MMSE score<21) was 4%; all were women.

### Co-variates of cognitive impairment

Upon univariate analysis of participants stratified by status of cognition, elderly having cognitive impairment were significantly older, lighter in weight, and had lower waist and hip circumference compared with elderly having no impairment. More elderly with cognitive impairment had no education and were living alone compared with those having no impairment (p<0.05). (Table 2) Self-reported health was significantly poor among those with cognitive impairment; so was depression (p<0.05). Elderly having cognitive impairment had significantly more difficulty in ADL, difficulty in mobility and were bedridden since past 6 months compared with those not having cognitive impairment (Table 2).

Logistic regression identified age, no schooling and bedridden for past 6 months as independent correlates in this population. Age per SD increase augmented the risk of cognitive impairment by nearly 3 folds (OR: 2.86, 95% CI: 1.19–6.90), no schooling by 8 folds (OR: 8.73, 95% CI: 1.32–57.47), while being bedridden for past six months or more was positively associated nearly 26 folds (OR: 25.95, 95% CI: 3.73–180.37) with cognitive impairment (Table 3).

## Discussion

The present study reported a cognitive impairment prevalence of 10% among Indian elders. The range of cognitive impairment has been reported between 3–19% in different parts of the world [32]. Our prevalence rates are lower compared with the US (22%, among > 71 years-olds) [33], Finland (26%, among 68–78 years-olds) [34], Spain (19%, among >65 years-olds) [35], China (30%, among >80 years-olds) [36] and Australia (33%, among 70–79 years-olds) [37]; much lower than Malaysia (68%) [38]; and higher than Iran (2.8%) [39].

A study on North Indian urban elderly reported a prevalence rate of 8.8%, [40] quite similar to ours. In a comparable South Indian study on elderly aged 65 years and above, the prevalence was reported to be 11.5%, conforming to our findings [41]. The Lucknow Elderly study reported a lower prevalence of 7.6%, using Cambridge Mental Disorders of the Elderly Examination-Revised tools in addition to MMSE for cognitive impairment measurement [21]. Elsewhere, a South African study reported cognitive impairment prevalence similar to ours (9%) among elderly aged 60 years and above. However, the tools used for cognitive function assessment were different [42]. Another study from Nigeria also reported 6.3% prevalence of cognitive impairment at baseline while at subsequent follow up after two years, 16% of the cognitively impaired developed dementia, while 58% remained cognitive impaired [43]. A systematic review of literature from Europe showed the prevalence to vary between 8–34%, the data however, included diverse ethnic populations [44].

Cognitive impairment in the present study was more among women compared with men. This difference of prevalence in genders has been reported by several other studies earlier [45,46], and attributed to survival bias among women who have mild cognitive impairment, when compared with men who have severe impairment and dementia. Some have also attributed disadvantage among elderly women due to reduction in estrogen levels leading to greater cognitive decline than men [47].

Higher age, no schooling and bedridden for past six months were the independent correlates of cognitive impairment in this study. Previous literature has shown a positive association of cognitive decline with increasing age, similar to our findings, which is physiologically plausible. Sengupta [40], and Maity [48] reported increasing age as a correlate for cognitive impairment among Indian elders. Several other studies have confirmed our finding of age being associated with cognitive decline [33,45,49]. Prior studies which reported age related cognitive impairment in older people, put forth the argument that older population already had underlying co-morbidities, like hypertension, diabetes mellitus, stroke, and cardiovascular disease that made them prone to get early onset dementia or cognitive impairment. Our data did not show a significant association of cognitive impairment with these variables. Others attributed cognitive impairment to age-related decreased brain volume, loss of myelin integrity, cortical thinning, and impaired secretion of neurotransmitters such as serotonin [50].

Illiteracy also had been shown to be significantly associated with cognitive impairment among elderly, similar to our finding. Khairiah [38] reported elderly with no education to have 6 fold odds for cognitive impairment among Malay population of mixed ethnicity. Illiteracy was earlier found to be independently associated among the North Indian elderly population as well [40]. Several others have also similarly shown illiteracy or no formal education to be associated with cognitive impairment and increased risk of dementia [49,51–53]. Prevalence of dementia decreases with increasing educational level, as observed in one systematic review which showed 2 folds dementia risk among subjects with low schooling (95% CI: 2.06–3.33) compared to subjects with higher educational level [54]. Brucki [55] has argued that illiteracy presents many factors that may be correlated to higher prevalence of dementia among illiterates including low cognitive reserve, poor control of cerebrovascular disease risk factors, difficulties in cognitive evaluation, and poor adaptation of neuropsychological tests. This review also highlighted that multiple factors related to illiteracy that included confounders and use of semantic relation for retention, among others were commonly found to be associated with poor performance on cognitive impairment measurement tools. Additionally it has been theorized that, having fewer years of formal education is associated with lower socioeconomic status [56], which in turn may increase one’s likelihood of experiencing poor nutrition and decrease their ability to afford health care or medical treatments, such as treatments for cardiovascular risk factors.

Our study reported independent association of bedridden status of elderly with cognitive decline. Amongst the very few studies that studied mobility status of participants, a study from Brazil reported bedridden or wheelchair-using elderly participants to have significant association with cognitive impairment, upon univariate analysis [57]. Another Brazilian study on assessment of institutionalized elderly people who had falls, reported the majority of them were vulnerable, with a significant dependence for activities of daily living (55.07%), impaired cognition (91.1%) and impaired physical mobility (88.4%) [58]. Inouye [59] designed a predictive model for functional decline in elderly that included cognitive impairment, functional decline and low social activity, that predicted 19% to 41% risk of hospitalization, thus underlining the strong association between these factors.

We reported a positive association with living single on univariate analysis. Ren [36] and Sengupta [40] have also shown cognitive decline to be associated with living single among elderly in China and India respectively. The DERIVA study, on the contrary did not find such association among Spanish elders [35]. Living single leads to faster cognitive decline because of its possible association with depression and lesser social engagements [60]. Moreover, neuro-pathological pathways for loneliness and cognitive impairment also exist [61]. These associations however, offer no information for clarifying whether the current situation is a consequence of or a risk factor for the development of cognitive decline.

We found an association of cognitive impairment with ADLs which was not an independent correlate upon logistic regression. Literature abounds on the association of ADLs and cognitive impairment since the ability to perform ADLs and IADLs is dependent upon cognitive (e.g., reasoning, planning), motor (e.g., balance, dexterity), and perceptual (including sensory) abilities [62]. There is also the important distinction of the individual’s ability to complete the task (physical and/ or cognitive ability) versus the ability to recognize that the task needs to be done without prompting (cognitive ability). Research has previously shown that the ability to complete ADLs is typically well preserved in mild-to-moderate cognitive impairment. Jefferson and colleagues [63] reported no differences in ADL functioning between individuals with mild cognitive impairment and those with no cognitive impairment. Helvik [64] reported associations of more severe dementia with poorer ADL functioning among prospectively followed nursing home residents. Chaves [65] however, showed significant association between mild cognitive impairment and difficulties in ADL among Brazilian elders aged 65 years and above. These associations may heavily be dependent on the assessment scales in mild cognitive impairment [66]. Moreover, propositions indicate a hierarchy in functional decline of ADLs as cognition worsens. Katz and colleagues [67] proposed that the basic activities learned last in early development were the first to decline as cognition deteriorated. An analysis of such effect of cognitive impairment was beyond the scope of this communication.

We reported significant correlation between cognitive impairment among elders and age, living single, poor self-reported health, depression, currently bedridden, having difficulty in moving around, having multiple aches and pains, and deceased mobility. Poor self-rated health, found associated to cognitive impairment in this study, has been shown to be positively associated earlier [68]. It has been argued that elders with poor cognitive functions are less alert and aware towards their general health and appearance, leading to multiple minor health problems among them.

Body weight, waist circumference and hip circumference were significantly higher among elders with no cognitive impairment compared with those having cognitive impairment upon univariate analysis. Our finding of lower BMI among the cognitive impaired supports the theory of“obesity paradox” reported by the very few studies earlier [69]. Prospective studies so far from American [70], Italian [71] and Korean elders [72] have shown this association between lower BMI and less cognitive function or decline. Indian elderly population is unique in having central obesity in spite of low BMI [73]. While association with high waist hip ratio has been reported significantly by many in Western populations [74], the reasons for inverse association of anthropometric parameters with cognitive impairment in our elderly remain unclear. However, the effect of our comparatively small sample size on the results cannot be ruled out. This rare finding of low BMI and other anthropometric parameters in Indian elderly population indicates that risk factors of cognitive impairment in Indian urbans are probably different and guides us to propose different risk reduction strategies for preventing cognitive impairment among elderly, with special focus on nutrition and muscle strength, both of which have proven benefits on cognition.

We also found an association of pain and cognitive impairment in this population, which disappeared on multivariate analysis. Association of generalised aches and pains with cognition is ill described, but it seems more though an indirect association as a consequence of increasing age and general debility where it may co-occur with or exacerbate cognitive impairment related to ageing associated brain changes [75]. Pain in older adults may lead to poorer cognitive function because the presence of pain may require attention and may compete for limited resources [76]. Lau-Ting [77], Brody [78], and Roy [79] have shown such association earlier among rural elderly and discussed that this association is accounted for by underestimation and under-treatment by health care teams. Additionally, it has also been theorized that most cognitive impairment studies used verbal self-report as a measure of pain, which could be impacted by both memory loss and dysphasia occurring commonly in dementia, leading to underreporting of pain [80].

We found significant association between decreased mobility and cognitive impairment. Inzitari [81] described temporal associations between cognitive impairment and mobility disability among Italian elderly. It has been theorized that cognitive abilities are crucial for ongoing planning, decision-making and monitoring of movements necessary for successful mobility [82].

### Strengths and Limitations

This study has some strength and significance. Firstly, it was based in a multidimensional theoretical model, enabling the identification of association of cognitive impairment with multiple factors including biological and psychological correlates, and geriatric syndromes such as pains and mobility disability. Secondly, it was one of the first population-based studies for cognitive impairment among urban older people in South India.

The main limitation was the small sample size that prevented us from generalising the findings with sufficient power. Post hoc power for prevalence however, was 90% (calculated using SPSS 21) using representative population findings from the US [33]. Some of the findings are not entirely comparable with international literature due to diverse yet unique ethnic and cultural setting in South India.

## Conclusion

Cognitive impairment prevalence was low in our urban elderly population than published reports from western countries. Prevalence among women was comparable with Western rates. Anthropometric measurements (weight, waist and hip circumference) on the lower side were associated with cognitive impairment indicating obesity paradox in our population. Increasing age, lack of schooling and bedridden for past six months were independently associated factors in our population. The increasing longevity as well as burden of geriatric problems may contribute to increase in cognitive impairment in near future. There is an urgent need of in-depth understanding of modifiable factors for cognitive impairment to halt the progression of cognitive impairment among elderly individuals.

## Figures and Tables

**Table 1:** Baseline demographic characteristics of the study population.

Characteristics	Men (% or mean + SD) (N = 52)	Women (% or mean + SD) (N = 48)	Total % or mean + SD) (N=100)	P value
Age (years) (mean)*	70.92 + 6.76	68.93 ± 7.30	69.97 ± 7.06	0.16
No schooling (%)	3.8	22.9	13.0	0.005
Marital status (living single) (%)	7.7	31.2	19.0	0.003
Joint family (%)	57.7	60.0	59.0	0.47
Height (cm) (mean)*	164.30 + 11.00	144.25 ± 15.06	154.58 ± 16.49	<0.001
Weight (kg) (mean)*	70.43 ± 11.88	62.33 ± 12.03	66.50 ± 12.57	0.001
Body mass index (kg/m^2^) (mean)*	26.15 ± 4.10	30.90 ± 8.18	28.45 ± 6.81	<0.001
Waist circumference (cm) (mean)*	93.58 ± 8.54	84.98 ± 11.31	89.40 ± 10.83	<0.001
Hip circumference (cm) (mean)*	101.73 ± 8.30	103.27 ± 10.67	102.4 ± 9.51	0.42
Currently working (%)	3.8	2.1	3.0	0.53
Cognitive function score (MMSE) (mean)*	27.94 ± 2.44	26.83 ± 3.44	27.41 ± 3.00	0.06

**Table 2:** Characteristics of men and women by status of cognitive function impairment.

Characteristics (correlates)	No Cognitive impairment (% or mean + SD) (n=90)	Cognitive impairment (% or mean + SD) (n= 10)	P value
**Univariate analysis of demographic characteristics in relation to cognitive impairment**			
Age (years) (mean)*	69.23 ± 6.72	76.60 ± 7.55	0.001
Height (cm) (mean)*	154.92 ± 16.48	151.51 ± 17.13	0.53
Weight (kg) (mean)*	67.59 ± 12.29	56.82 ± 11.29	0.009
Body mass index (kg/m^2^) (mean)*	28.80 ± 6.68	25.33 ± 7.55	0.12
Waist circumference (cm) (mean)*	90.24 ± 10.56	82.00 ± 10.98	0.02
Hip circumference (cm) (mean)	103.21 ± 9.22	95.90 ± 9.99	0.02
Marital status (living single) (%)	15.0	50.0	0.02
No schooling (%)	8.9	50.0	0.003
Joint family (%)	38.9	60.0	0.17
Not Currently working (%)	96.7	100	0.72
**Univariate analysis of medical history and comorbidities in relation to cognitive impairment**			
Smoking (%)	8.9	20.0	0.26
Alcohol (%)	16.7	0.0	0.18
Self-reported health status (poor) (%)	56.7	90.0	0.03
Knee pain (self-reported) (%)	36.7	60.0	0.13
Poor Vision (%)	22.2	30.0	0.41
Poor Hearing (%)	16.7	40.0	0.09
Osteoarthritis (self-reported) (%)	42.2	60.0	0.22
Stroke (self-reported) (%)	4.4	20.0	0.10
Hypertension (self-reported) (%)	58.9	70.3	0.37
Hypertension (self-reported / measured high blood pressure) (%)	68.9	70.0	0.62
Myocardial Infarction (self-reported) (%)	22.2	10.0	0.33
Angina (self-reported) (%)	24.2	10.0	0.27
Cardiovascular disease (all) (%)	35.5	20.0	0.27
Asthma (self-reported) (%)	6.7	20.0	0.18
Diabetes (%)	41.1	50.0	0.41
Depression (GDS-15 point scale) (> 5) (%)	20.0	50.0	0.04
Systolic Blood pressure (mmHg)(mean)*	134.36 ± 19.99	139.00 ± 13.83	0.47
Diastolic blood pressure (mmHg)(mean)*	78.26 ± 10.47	72.00 ± 5.27	0.06
**Univariate analysis of Functional disabilities in relation to cognitive impairment**			
Activities of Daily Living (ADL) (% with difficulty)	31.1	70.0	0.02
Bed ridden (past 6 months) (%)	10.0	60.0	0.001
Difficulty in moving around (across room) (%)	27.8	70.0	0.01
Body aches or pains (obstructing daily life work) (%)	23.3	50.0	0.07
Difficulty in self-care (%)	6.0	20.7	0.18
Low physical activity (no exercise/no sports) (%)	44.4	50.0	0.49
Quality of sleep (PSQI) (% with poor sleep quality)	34.4	30.0	0.50

**Table 3:** Logistic regression analysis for predicting independent risk factors of cognitive impairment.

Risk factors	Odds ratio (OR)	95% Confidence Interval (CI)	
		Lower	Upper
Age (per 1 SD increase) *	2.86	1.19	6.90
No schooling*	8.73	1.32	57.47
Bed ridden (past 6 months)**	25.95	3.73	180.37

Variables in the model: Sex, age (per 1 SD increase), weight (per 1 SD increase), waist circumference (per 1 SD increase), hip circumference (per 1 SD increase), marital status, education, self-reported health status, depression, activities of daily living (ADL) and bed ridden.

* p<0.05;

** p=0.001, *t test statistics
